# The potential of plant microbiota in reducing postharvest food loss

**DOI:** 10.1111/1751-7915.13252

**Published:** 2018-03-26

**Authors:** Franziska Buchholz, Tanja Kostić, Angela Sessitsch, Birgit Mitter

**Affiliations:** ^1^ AIT Austrian Institute of Technology GmbH Center for Health & Bioresources, Bioresources Konrad‐Lorenz‐Strasse 24 A‐3430 Tulln Austria

## Abstract

The role of the plant microbiota in plant establishment, growth and health is well studied, but the dynamics of postharvest crop microbiota and its role in postharvest crop quality are largely unexplored, although food loss is an enormous issue worldwide. The microbiota might be especially important during crop storage by either preventing or favouring rots, or quality loss due to, for example, sprouting, saccharification, water loss or spoilage. We need more research on plant–microbe interactions in postharvest crops to be in future able to provide microbial solutions for plant production along the whole food chain from field to fork.

Food waste and loss is an enormous issue worldwide. The FAO states that ‘Hunger is still one of the most urgent development challenges, yet the world is producing more than enough food.’, but a vast amount of what is produced gets lost on its way from the field to the consumer (FAO, 2015). According to the FAO, about 30% of cereals, 20% of dairy products, 35% of fish and seafood, 45% of fruits and vegetables, 20% of meat, 20% of oilseed and pulses and 45% of roots and tubers are lost or wasted. This is one‐third of all the food produced for human consumption (FAO, 2015). However, instead of decreasing postharvest losses to ensure food supply, the efforts in recent years focused at increasing food production. This is also reflected in plant microbiome research, where the focus is clearly on the exploitation of plant–microbe interactions for improved plant productivity.

Postharvest food loss (PHL) is defined as measurable qualitative and quantitative food loss along the supply chain, starting at the time of harvest till its consumption or other end uses (Hodges *et al*., 2010). Food losses can either be the result of a direct quantitative loss, for example, during the process of harvest or transport or arise indirectly due to losses in crop quality such as undesired sprouting or water loss (Aulakh and Regmi, 2013). Postharvest food loss consists of many factors, which have not changed much in the last 40 years and so postharvest food loss in general has not changed much. As an example, in 1975, the postharvest losses of rice in the Philippines were estimated between 10 and 37% (Bourne, 1977). In 2010, exactly the same values were assessed (Parfitt *et al*., 2010). Even if the causes and extent of postharvest food loss vary across countries due to differences in economic development, this example demonstrates that our knowledge about postharvest handling of crops did not improve much in decades. Figure 1 illustrates the main reasons for food loss along the supply chain and is based on ‘The food pipeline’ published by Bourne in 1977 (Bourne, 1977). Food loss is highest during storage due to pathogens (insects, bacteria and moulds), environmental conditions (e.g. rain, humidity, heat and frost), sprouting and quality loss (rancidity, water loss and saccharification) or animals (rodents and birds). Recently published data for Switzerland have shown that about 53%–55% of the initial fresh potato production and 41%–46% of the initial processing potato production are lost mostly due to pathogen infection, saccharification, water loss of tubers and early sprouting during storage (Willersinn et al., 2015).

**Figure 1:**
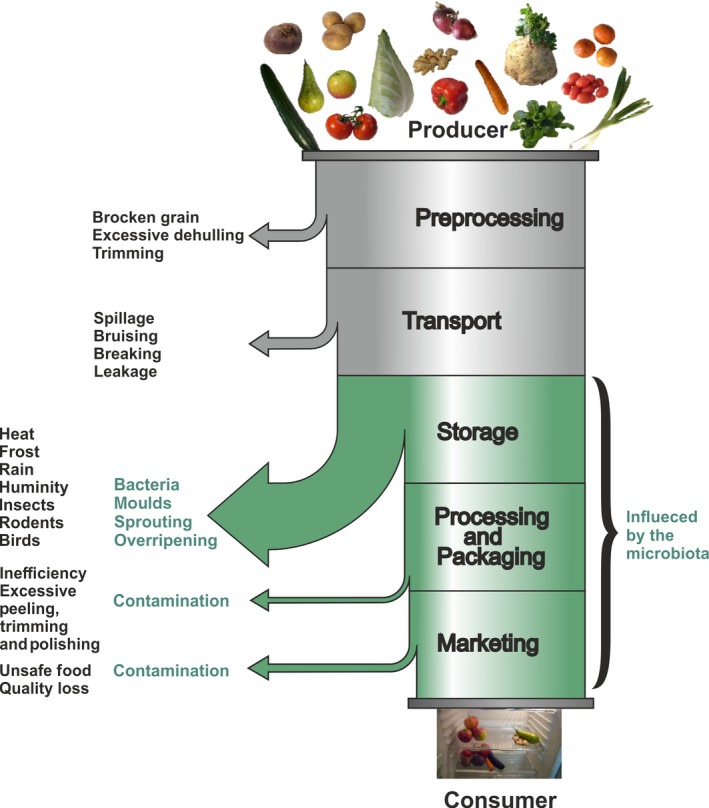
Along the food pipeline that takes the food from the field to the table, there are many opportunities for food to be lost with the result that much of the food that has been produced, never reaches the consumer for whom it was intended (adapted from Bourne, 1977). The size of the arrows reflects the relative food loss at each postharvest handling step.

However, how does the postharvest food loss relate to plant microbiota and which role could the microbiota play in reducing losses? If we take a closer look at the food pipeline in Figure 1, it becomes clear that microorganisms may predominantly influence the storage of crops. However, while the microbiota of plants in vegetative growth and its role in plant health and growth are well studied and agro‐industry is making great achievements in the development of microbial applications for increasing plant productivity, the postharvest microbiota of crops and its impact on storage stability is largely unexplored.

Pathogen‐induced decay is certainly the most obvious reason for postharvest crop loss caused by microbial activity (bacteria and moulds). Plant microbiomes consist of complex communities of potentially mutualistic, commensal and pathogenic microbes colonizing the same niches in plants, including, for example, grains (Baker and Smith, 1966; Sheibani‐Tezerji *et al*., 2015). Moreover, a clear distinction between plant‐beneficial or harmful microorganisms is not always possible (Hentschel *et al*., 2000). The outcome of plant–microbe interactions depends on biotic and abiotic environmental factors but also the manifold interactions between members of the plant microbiota, including pathogens, strongly influence the phenotype of plant–microbe interactions (Brader *et al*., 2017). Often disease outbreak in plants correlates with shifts in the microbiome composition, resulting in a microbial dysbiosis and a response of specific microbes, which can act as antagonists or synergists towards plant pathogens (Berg *et al*., 2017). Only a few studies have investigated the dynamics of the microbiota during postharvest disease development. Kõiv *et al*. (2015) described changes in the bacterial community composition of potato tubers in response to infection with the soft rot pathogen *Pectobacteriumatrosepticum*. In conclusion, the study showed that pathogenicity is triggered by the pathogen, but the endophyte community strongly contributes to the development of the disease (Kõiv *et al*., 2015). More recently, Liebe *et al*. (2016) reported shifts in the fungal and oomycete community composition associated with storage soft rot development in different sugar beet hybrids from different environments stored at different temperatures. Interestingly, while the genotypes showed differences in the susceptibility to soft rot, the shifts in the microbial community of sugar beet were genotype independent. The authors suggested an unspecific resistance mechanism slowing down the spread of pathogens in more resistant genotypes but not preventing infection (Liebe *et al*., 2016). However, the study focused on fungi and oomycetes and the dynamics of bacteria community during storage soft rot development of sugar beet and its putative role in disease expression remains elusive. In summary, pathogen infestation and spoilage may often not be caused by a single organism but is likely to result from the interplay of individual members of the microbial community in crops. On the other hand, stored plant organs such as seeds may contain bacteria with antagonistic activity against plant pathogens (Fürnkranz *et al*., 2012) with the potential to protect plants not only in the field but also postharvest (Glassner *et al*., 2015). Furthermore, it was suggested that the diversity of a microbial community in plants determines pathogen establishment (Berg *et al*., 2017). The seed microbiome of oilseed rape, for example, is cultivar‐specific and cultivars hosting a higher indigenous microbial diversity showed better resistance towards colonization by pathogenic microorganisms (Rybakova *et al*., 2017).

The activity of microorganisms could also affect sprouting, ripening and quality losses such as moisture loss or saccharification during crop storage. Premature sprouting of tubers or onions is a problem during storage, especially in industrialized countries, where consumers want to buy plant produce that does not show signs of sprouting, all over the year. In potato, for example, sprouting is a complex physiological process involving usage of storage reserve metabolites such as carbohydrates and proteins (Aksenova *et al*., 2013). Recently, it was shown that improved seed germination and seedling growth of *A. bifolim* in response to inoculation with endophytic bacteria were coupled with increased storage reserve mobilization and the degradation of proteins, lipids and sucrose (Zhu *et al*., 2017). Phytohormones play a key role in the regulation of sprouting – cytokinins and indole‐3‐acetic acid signalling induce the onset of sprouts, and gibberellin stimulates sprout growth (Aksenova *et al*., 2013). Many plant‐associated bacteria are able to produce plant hormone‐like metabolites, and the role of auxins, cytokinins and gibberellins produced by bacteria in plant growth regulation is well documented (e.g. Steenhoudt and Vanderleyden, 2000; Patten and Glick, 2002; Spaepen *et al*., 2007; Ortíz‐Castro *et al*., 2008; Cassán *et al*., 2009; Lambrecht *et al*., 2000). Slininger *et al*. (2003) described an inhibitory effect of six bacterial strains on potato sprouting. The bacteria, which were originally studied for their antagonistic activity towards potato rot fungi, significantly reduced sprouting in postharvest tubers of the cultivar Russet Burbank during storage (Slininger *et al*., 2004). Those bacteria exhibiting sprout inhibitory effects were found to produce the plant hormone indole‐3‐acetic acid (Slininger *et al*., 2003). Considering all these findings, it appears likely that the microbiome composition in storage crops influences dormancy duration.

Of course, sprouting of crops could be prevented easily by storing at low temperatures (4°C). This, however, causes enormous energy consumption and favours quality loss because of ‘cold‐induced sweetening’ (different from senescent sweetening), a phenomenon which is typically observed in stored starchy crops such as potato or cereals and was already documented in 1882 (Müller‐Thurgau, 1882). The sweeting is based on saccharification of starch, which means that starch is converted to the reducing sugars glucose and fructose. This process is favoured at lower storage temperatures (Kumar *et al*., 2004). The actual problems arise during processing of starchy crops, such as frying, roasting or baking, when the reducing sugars react with free amino acids (asparagine) to acrylamide (Mottram *et al*., 2002), a potentially carcinogenic substance. Proposed strategies to limit the accumulation of reducing sugars in tubers include preventing sucrose accumulation and preventing sucrose conversion into reducing sugars (Dale and Bradshaw, 2003). Many bacteria and fungi possess enzymes such as α‐amylases, which hydrolyze starch molecules into polymers composed of glucose units (De Souza and De Oliveira Magalhães, 2010) and so the activity of plant‐associated microorganisms could play a role in the accumulation of reducing sugars in plants. Whether modulating the microbiome with the aim of suppressing amylase activity could contribute to solving this problem remains elusive but is worth investigating.

Moisture loss and consequently weight loss through transpiration is one of the main reasons for optical quality penalties with vegetables during storage. Drought stress during plant growth results in transpiration reduction and this continues postharvest (Graf and Herppich, 2012). It is well known that inoculation with certain plant‐beneficial bacteria can minimize symptoms of drought stress in plants and one of the effects is that the water content in plant tissue remains higher during drought stress periods than in non‐inoculated control plants (Naveed *et al*., 2014). The application of microbial inoculants that reduce drought stress on the field could therefore also have positive effects on water content of crops during storage.

Microorganisms could also play a role in the ripening process of crop plants. About 20% of postharvest loss of fruits and vegetables is attributed to prolonged climacteric ripening that leads to senescence, apoptosis, lesions, spotting, bruising, infection and spoilage (Perry and Williams, 2014). During ripening, climacteric plants release various volatile compounds, including ethylene, a hydrocarbon, which can be metabolized by many microorganisms, thereby affecting plant development (Teranishi and Saima, 1993; Elsgaard and Andersen, 1998). However, fungi and bacteria, which are living inside fruits and vegetables, can also produce ethylene themselves (Freebairn and Buddenhagen, 1964; Digiacomo *et al*., 2014). Besides direct modulation of ethylene levels in plants, microorganisms can also indirectly affect ethylene in plants. Plants produce ethylene from 1‐aminocyclopropane‐1‐carboxylate (ACC) via ACC oxidase activity (Burg and Burg, 1962). Many bacteria in soil and plants possess ACC deaminase activity, which cleaves ACC to 2‐oxobutyrate and ammonia and can contribute to ethylene balancing in plants (Glick, 2005). An alternative role of ethylene in microbial‐driven modulation of ripening was introduced by a recent study exploring the potential of a *Rhodococcus rhodochrous* strain to delay ripening in different species of climacteric fruits (Pierce *et al*., 2011). In this case, the proposed mechanism is that ethylene‐induced nitrile hydratase and/or nitrilase activity in the bacterium, which resulted in maintenance of fruit firmness and reduced spotting and spoilage (Perry, 2011). More recently, the same authors demonstrated that application of ethylene gas to soil induced changes in the soil microbial activity, resulting in the release of a nitrile compound, which negatively affected climacteric ripening of organically grown bananas and peaches (Perry and Williams, 2014). This is a good example for the impact of the soil microbiota on crop quality and subsequent storage properties.

The soil, in which crop plants have been grown, could generally affect storage stability of crops, that is, via the nutrient and water content but also via the soil microbiota. The soil is the main reservoir for the recruitment of microorganisms colonizing plants. Bacteria and fungi, attracted by root exudates migrate to the rhizosphere, further into the rhizoplane and some of them may also invade and colonized inner plant tissue (Compant *et al*., 2010). The soil microbiota is therefore also the main driver in the composition of the crop‐associated microbiota. However, the impact of the soil microbial community composition on storage stability of crops is still a black box.

Our literature search has clearly revealed that the potential of the plant microbiome to influence postharvest losses is largely unexplored. Of course, there might be issues causing postharvest losses, which are independent from the microbiome, for example, a mouse might eat an apple no matter how the microbiome looks like. However, exploring the role of the plant microbiota in storage stability of crops could in future ensure food supply and food quality by employing microbial‐based solutions from the field to the fork.

## Conflict of interest

Authors have no conflict of interest to declare.
